# The Development of Surface-Modified Liposomes as an Intranasal Delivery System for Group A Streptococcus Vaccines

**DOI:** 10.3390/vaccines11020305

**Published:** 2023-01-30

**Authors:** Jieru Yang, Jennifer C. Boer, Mattaka Khongkow, Sarunya Phunpee, Zeinab G. Khalil, Sahra Bashiri, Cyril Deceneux, Georgia Goodchild, Waleed M. Hussein, Robert J. Capon, Uracha Ruktanonchai, Magdalena Plebanski, Istvan Toth, Mariusz Skwarczynski

**Affiliations:** 1 School of Chemistry and Molecular Biosciences, The University of Queensland, St. Lucia, QLD 4072, Australia; 2 School of Health and Biomedical Sciences, RMIT University, Bundoora, Melbourne, VIC 3083, Australia; 3 National Nanotechnology Center (NANOTEC), National Science and Technology Development Agency (NSTDA), 111 Thailand Science Park, Phahonyothin Road, Klong 1, Pathumthani 12120, Thailand; 4 Institute for Molecular Bioscience, The University of Queensland, St. Lucia, QLD 4072, Australia; 5 School of Pharmacy, The University of Queensland, Woolloongabba, QLD 4102, Australia

**Keywords:** group A Streptococcus, oleoyl-quaternized chitosan, adjuvant, intranasal vaccine, multilamellar liposome, cell-penetrating peptide

## Abstract

Intranasal vaccine administration can overcome the disadvantages of injectable vaccines and present greater efficiency for mass immunization. However, the development of intranasal vaccines is challenged by poor mucosal immunogenicity of antigens and the limited availability of mucosal adjuvants. Here, we examined a number of self-adjuvanting liposomal systems for intranasal delivery of lipopeptide vaccine against group A Streptococcus (GAS). Among them, two liposome formulations bearing lipidated cell-penetrating peptide KALA and a new lipidated chitosan derivative (oleoyl-quaternized chitosan, OTMC) stimulated high systemic antibody titers in outbred mice. The antibodies were fully functional and were able to kill GAS bacteria. Importantly, OTMC was far more effective at stimulating antibody production than the classical immune-stimulating trimethyl chitosan formulation. In a simple physical mixture, OTMC also enhanced the immune responses of the tested vaccine, without the need for a liposome delivery system. The adjuvanting capacity of OTMC was further confirmed by its ability to stimulate cytokine production by dendritic cells. Thus, we discovered a new immune stimulant with promising properties for mucosal vaccine development.

## 1. Introduction

Intranasal delivery is one of the most advantageous routes for vaccine administration [1,2,3]. Mucosal surfaces are well-vascularized, enabling rapid antigen absorption into the lymphatic system. The activity of proteolytic enzymes in the nasal cavity, which can potentially destroy antigens, is low in comparison to the oral delivery pathway. Intranasal immunization (a) can trigger systemic immune responses; (b) is needle-free and has high patient compliance; (c) can be self-administered and, therefore, the cost of vaccine administration can be greatly reduced (no need for specialized personnel); and (d) has greater capacity for mass immunizations. Furthermore, nasal-associated lymphoid tissue, the main tissue involved in intranasal immunity, is similar in humans and rodents, which greatly simplifies the translation of animal study into clinical trials. Two intranasally delivered vaccines (live-attenuated virus-based) have already been approved and licensed for influenza (FluMist/Fluenz™ and Nasovac™). However, before any vaccine can be approved for intranasal delivery, it must overcome a variety of challenges. These include (a) fast mucosal clearance and, therefore, limited interaction time of a vaccine with the mucosal surface; (b) poor mucosal permeability; (c) efficacy even with the limited dosage that can be administered via the nose; and (d) a lack of effective approved mucosal adjuvants (immune stimulators). These limitations can all lead to suboptimal vaccine efficacy. Consequently, the need exists for an effective mucosal vaccine self-adjuvanting delivery system. Furthermore, self-adjuvanting delivery systems provide additional advantages by circumventing the requirement of toxic adjuvants, such as complete Freund’s adjuvant (CFA) [4,5,6], while still inducing potent immune responses.

**Figure 1:**
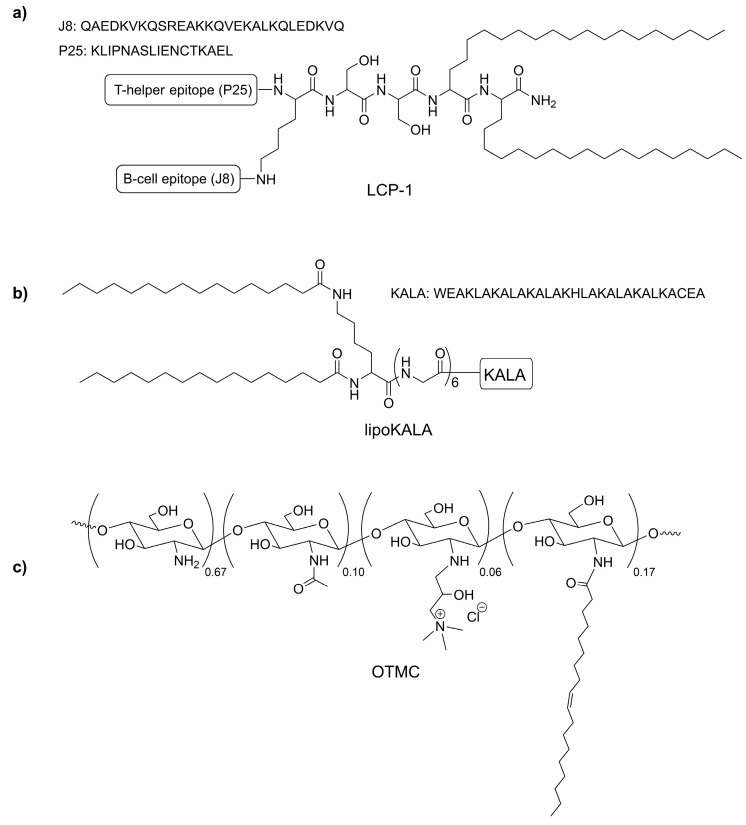
The chemical structure of (**a**) LCP−1, (**b**) lipoKALA, and (**c**) OTMC.

Group A Streptococcus (*Streptococcus pyogenes*, GAS) is a gram-positive bacteria that causes a variety of diseases, from common pharyngitis (strep throat) to deadly rheumatic heart disease (RHD) [7]. RHD, alone, results in hundreds of thousands of deaths per year, worldwide [8]. There is no vaccine available to prevent this infection. Importantly, the traditional live-attenuated pathogen-based strategy cannot be used for GAS vaccine development, as the whole bacterium induces autoimmune reactions. Thus, a major virulent factor of GAS, M protein, was selected as the most promising vaccine antigen. Since similarities of M protein sequence with human proteins were detected, the development of the GAS vaccine has been purely focused on short peptide epitopes derived from GAS proteins. Significantly, all clinically tested vaccine candidates in the last two decades have been composed of peptide epitopes derived from M-protein [9]. Among them, J8 peptide (QAEDKVKQSREAKKQVEKALKQLEDKVQ) is the only conserved epitope recognized by a variety of GAS strains that reached clinical trials [10]. Thus, we selected J8 as an antigen for mucosal vaccine development and investigated a variety of intranasal delivery platforms for one of our lead GAS vaccine candidates, LCP−1 (Figure 1a) [11,12].

Liposome-based systems are one the most popular intranasal delivery platforms [2,13,14], especially as the liposomes’ immunological properties can be modified by polyelectrolyte-based coatings [15,16]. These coatings can stabilize liposomes, and more effectively protect encapsulated antigens and improve their mucoadhesive properties. For example, we have demonstrated that the immunogenicity of liposomes bearing lipopeptide-based vaccines was enhanced by coating liposomes with alginate and trimethyl chitosan (TMC) [17]. Moreover, when such vaccine was coated with dextran and TMC and used for intranasal immunization of mice, higher antibody titers were observed compared to antigen delivered with a commercial mucosal adjuvant, cholera toxin subunit B [18]. Interestingly, once negatively charged dextran was converted to its cationic derivative, diethylaminoethyl(DEAE)-dextran, it provided adjuvanting activity on its own [19,20]. We also recently showed that cell-penetrating peptides (CPPs), such as lipidated KALA (WEAKLAKALAKALAKHLAKALAKALKACEA) and polyethylenimine (PEI), can improve vaccine efficacy upon intranasal immunization (Figure 1b) [21,22]. While TMC is a widely investigated mucoadhesive polymer, Ruktanonchai and co-workers recently demonstrated that oleoyl-quaternized chitosan (OTMC, Figure 1c) also has mucoadhesive properties [23]. Therefore, we hypothesized that OTMC may also improve the immunogenicity of mucosal vaccines.

**Figure 2:**
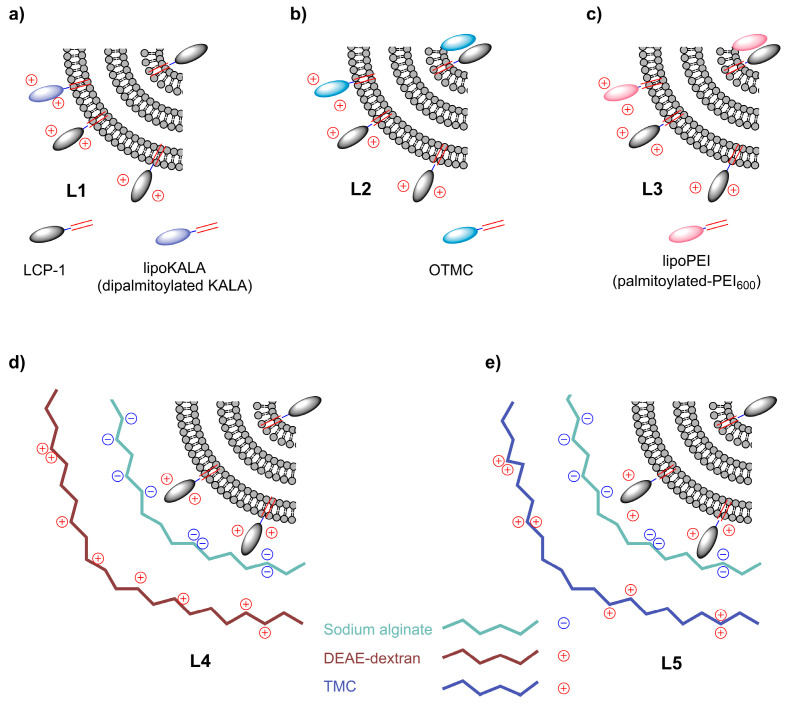
The multilamellar liposome−based LCP−1 vaccine delivery system bearing immunity enhancers, (**a**) lipoKALA (**L1**), (**b**) OTMC (**L2**), (**c**) lipoPEI (**L3**), (**d**) DEAE-dextran (**L4**), and (**e**) TMC (**L5**).

As a leading vaccine candidate against GAS, lipopeptide LCP−1 was selected for this study [11,24]. LCP−1 carries universal P25 T-helper epitope, J8, GAS M protein-derived B-cell epitope, and two lipidic moieties (Figure 1). Here, we designed several delivery systems for the LCP−1 vaccine to determine which has the greatest capacity to induce strong and effective antibody responses following intranasal immunization. These included five liposome-based vaccine candidates: LCP−1 encapsulated into liposomes carrying lipoKALA peptide (**L1**); [21] OTMC (**L2**); lipoPEI (**L3**); [22] and LCP−1 coated with alginate/DEAE-dextran (**L4**); or alginate/TMC (**L5**) (Figure 2). Moreover, three corresponding physical mixtures were also investigated: LCP−1/OTMC; LCP−1/DEAE-dextran; and LCP−1/TMC, which contained equivalent amounts of LCP−1 and polymers to the liposomal formulations. The vaccine candidates were evaluated in outbred mice following intranasal administration. A common key feature that defines many vaccine adjuvants is an ability to stimulate immune-stimulatory cytokines from the main immune-activating cell of the immune system, the dendritic cell (DC) [25]. Indeed, DC are uniquely capable of priming the immune system to recognize new pathogens, given their ability to endocytose, process and present antigens to lymphocytes, and release secondary signals, such as pro-inflammatory cytokines, which further result in their production and activation. Therefore, the ability of vaccines to stimulate cytokine production by DC has also been evaluated.

## 2. Materials and Methods

All chemicals used in this study were analytical-grade or equivalent, unless stated otherwise. Protected Fmoc/Boc-amino acids were obtained from Novabiochem (Läufelfingen, Switzerland). 1-[Bis(dimethylamino)methylene]-1*H*-1,2,3-triazolo[4,5-b]pyridinium 3-oxid hexafluorophosphate (HATU) was purchased from Mimotopes (Melbourne, Australia). *p*-MBHA·HCl resin was purchased from Peptides International (Louisville, KY, USA). Rink amide 4-methylbenzhydrylamine (MBHA) resin was purchased from Novabiochem (Hohenbrunn, Germany). Methanol, dichloromethane (DCM), *N,N′*-dimethylformamide (DMF), chloroform, *N,N*-diisopropylethylamine (DIPEA), HPLC-grade acetonitrile, trifluoroacetic acid (TFA), and piperidine were purchased from Merck (Hohenbrunn, Germany). Triisopropylsilane (TIPS), copper wire, phenylmethylsulfonyl fluoride (PMSF), and phosphate-buffered saline (PBS) tablets were purchased from Gibco (Paisly, UK). *O*-phenylenediamine dihydrochloride (OPD) was purchased from SIGMAFAST^TM^. Goat anti-mouse IgG conjugated to horseradish peroxidase and its substrate were purchased from Bio-Rad (Hercules, CA, USA). Dipalmitoylphosphatidylcholine (DPPC), cholesterol (CH), an Avanti mini extruder, PC membranes, and filter supports were purchased from Avanti (Alabaster, AL, USA).

Analytical reverse-phase high-performance liquid chromatography (RP-HPLC) was performed on a Shimadzu LCMS-2020 instrument (Kyoto, Japan) with a Vydac analytical C-4 (214TP; 10 µm, 250 × 4.6 mm) or C-18 column (218TP; 10 µm, 250 × 4.6 mm) at a flow rate of 1 mL/min. Detection was conducted at 214 nm. Preparative RP-HPLC was performed on a Shimadzu instrument using either a Vydac or Altima preparative C-18 column (218TP; 10 mm, 250 × 22 mm) and C-4 column (214TP; 10 mm, 250 × 22 mm), or a semi-preparative column in linear gradient mode using a flow rate of 10–20 mL/min. Detection was conducted at 214 nm. Compounds were synthesized as previously reported: LCP−1 [26], lipoKALA [21], OTMC [23], lipoPEI [22], and TMC (degree of quaternization 65%) [27].

### 2.1. Preparation of LCP−1-Loaded Multilamellar Liposomes

All liposomal formulations were prepared to contain 1 mg/mL of LCP−1.

Liposomes **L1** were formulated with DPPC, CH, LCP−1, and lipoKALA at a molar ratio of 2:1:0.05:0.01. DPPC (4 mg in 1 mL chloroform), CH (1.05 mg in 1 mL chloroform), LCP−1 (1 mg in 0.5 mL methanol), and lipoKALA (0.135 mg in 0.5 mL methanol) were mixed together in a 5 mL round bottom flask. The solvents were very slowly removed under reduced pressure to produce a dry lipid film. The flask was stored under a high vacuum (in a freeze dryer) overnight to remove residual solvent. The formed film was rehydrated with Milli-Q water (1 mL) to produce multilamellar liposomes.

Liposomes **L2** and **L3** were formulated in an identical manner to **L1**, but with DPPC, CH, LCP−1, and OTMC at a molar ratio of 2:1:0.05:0.01 (**L2**); and DPPC, CH, LCP−1, and lipoPEI at a molar ratio of 2:1:0.05:0.01 (**L3**).

Liposomes **L4** and **L5** were formulated with DPPC, CH, and LCP−1 at a molar ratio of 2:1:0.05 in a similar manner to **L1**, except an additional coating was performed. The liposome coating procedure was performed after thin-film hydration and was based on electrostatic interactions. The optimal amount of polymer in the coating (sodium alginate, DEAE-dextran, and TMC) was determined based on the least amount required for full-surface coating, as detected by changes in zeta potential (Supplementary Figure S1). The rehydrated liposome solution (1 mL) was split into 10 individual vials (100 µL liposome solution in each, containing 100 µg LCP−1), while three polymer stock solutions (sodium alginate, TMC, and DEAE-dextran) were prepared to a 3 mg/mL concentration in Milli-Q water. Stock solutions were stirred for 2 h prior to the coating process. Various amounts of alginate stock solution (0, 5, 10, 20, 30, 40, 60, 65, 70, 75, 80, 100, or 200 µg of alginate) were added drop-by-drop to 100 µL liposome solutions and incubated for 1 h with gentle stirring at room temperature. The zeta potential of each solution was monitored by DLS, a stable negative zeta potential indicated that the coating was complete. The saturation quantity of sodium alginate to coat 100 µL of liposome solution (containing 100 µg LCP−1) was identified as 75 µg. Rehydrated liposome solution (1 mL) with 750 µg of coating was prepared and split into 10 individual vials. DEAE-dextran stock solution (0, 80, 110, 115, 125, 130, 150, 200, or 240 µg) was added drop-by-drop to individual alginate-coated liposome solutions (containing 100 µg LCP−1); the solutions were then incubated for 1 h with gentle stirring at room temperature. The optimal amount of DEAE-dextran coating was identified as 125 µg/100 µL liposomes (containing 100 µg LCP−1); this was used to produce **L4**. The amount of TMC for coating (150 µg/100 µL) of **L5** was determined in the same way.

### 2.2. Characterization of Vaccine Candidates by Dynamic Light Scattering

Average particle size, zeta potential, and the polydispersity index (PDI) were determined by dynamic light scattering (DLS) at a back-scattering angle of 173° at 25 °C in folded capillary cuvettes, using a Zetasizer Nano ZP instrument (Malvern, UK) with Malvern Zetasizer Analyser 6.2 software. The formulations were tested at 0.1 mg/mL concentration.

### 2.3. Immunization Study

Outbred female Swiss (CD-1) mice (9–10 weeks old) obtained from the Animal Resource Centre (Perth, Western Australia) were used for the immunization study. Mice were immunized intranasally with 30 μL (15 μL/nare) of PBS (negative control group), **L1**, **L2**, **L3**, **L4**, or **L5** (five mice per group). Each liposomal formulation, **L1–L5**, contained 30 µg of LCP−1. Mice were also immunized with physical mixtures of LCP−1/OTMC (containing 30 µg of LCP−1, and 45 µg of OTMC), LCP−1/DEAE-dextran (30 µg of LCP−1, and 37.5 µg of DEAE-dextran), LCP−1/TMC (30 µg of LCP−1, and 45 µg of TMC), and LCP−1 alone (30 µg). Three boosts were performed on days 14, 28, and 42. Blood was collected via tail bleed on days −1, 13, 27, and 41 and by cardiac puncture on day 52. The clear supernatant serum was collected after centrifugation for 10 min at 956× *g* (3600 rpm). Serum samples were stored at −80 °C.

### 2.4. Determination of IgG Titres

ELISA was used to measure J8-specific IgG antibody titers, as previously described [21]. Briefly, plates were coated with J8 (50 μg/plate) in carbonate coating buffer and then blocked with a 5% skim milk/PBS-Tween 20 buffer. Serial dilutions of two-fold serum samples were implemented in a 0.5% skim milk/PBS-Tween 20 buffer, with an initial serum dilution of 1:200. Horseradish-conjugated goat anti-mouse IgG (H + L) was used as a secondary antibody and O-phenylenediamine as substrate. An antibody titer was described as the lowest concentration producing an absorbance (at 450 nm) that was higher than 3 standard deviations (STD) above the average of serum from PBS-immunized mice (negative control).

### 2.5. Opsonization Assays

Opsonization assays were performed as described previously [28], using clinical isolates of D3840 (nasopharynx swab) and GC2203 (wound swab) donated by the Princess Alexandra Hospital (Brisbane, Australia). Briefly, bacterial isolates were streaked onto Todd–Hewitt broth supplemented with 5% yeast extract agar plates, then incubated for 24 h at 37°C. Single colonies were transferred to Todd–Hewitt broth (5 mL) supplemented with 5% yeast extract and incubated for 24 h at 37 °C to produce approximately 4.6 × 10^6^ colony-forming units (CFU)/mL. The culture was serially diluted (×100) in PBS. Aliquots (10 μL) were mixed with horse blood (80 μL) and heat inactivated sera (10 μL, inactivated using a water bath at 50 °C for 15 min). Bacteria were incubated with the sera in a 96-well plate for 3 h at 37 °C. Bacterial survival was examined by plating a 10 μL aliquot of the culture material onto Todd–Hewitt agar plates supplemented with 5% horse blood and 5% yeast extract. Plates were incubated for 24 h at 37 °C and colonies were counted as CFU. Antibody opsonic activity (%) was calculated as (1 − [CFU in the presence of immunized mouse serum]/[mean CFU in the presence of untreated wells]) × 100%. Assays were performed from two independent cultures, in duplicate.

### 2.6. Ex vivo Cytokine Profiling in Dendritic Cells

Bone marrow-derived dendritic cells (BMDCs) were obtained from C57BL/6 mice (6–8 week-old) in triplicate, for each condition. Briefly, mice were culled by CO_2_ asphyxiation. The femur and tibia of both legs were extracted and soaked in ethanol (70%) for 1 min, then washed and soaked in sterile Roswell Park Memorial Institute (RPMI) complete media (supplemented with 10% fetal bovine serum (FBS), 20 mM HEPES, 2 mM L-glutamine, 0.1 mM 2-mercaptoethanol, 100 μg of streptomycin and 100 units/mL penicillin; complete media (CM)). To extract bone marrow cells, bones were flushed and cells were then dissociated with a pipette, filtered through a cell strainer (100 μm, Millipore, Billerica, MA, USA) into a centrifuge tube (10 mL) and centrifuged at 372× *g* at room temperature for 5 min. The supernatant was removed and the cells were re-suspended in ammonium-chloride-potassium (ACK) lysis buffer (1 mL) for 1 min for erythrocyte lysis. The lysis buffer reaction was stopped with CM (9 mL). The cells were centrifuged again (372× *g*) at room temperature for 5 min, the supernatant was removed, and bone marrow cells were re-suspended in CM (10 mL). The bone marrow cells were then adjusted to 5 × 10^5^ cells/mL in CM, plated in 6-well plates (Corning) and granulocyte and monocyte colony-stimulating factor (GM-CSF) (PeproTech, Rocky Hill, NJ, USA) was added to the cell suspension to reach a concentration of 10 ng/mL. Bone marrow cells were adjusted to a concentration of 5 × 10^5^ cells/mL in CM and incubated for 3 days in 5% CO_2_ at 37 °C. On day 3, the cells were incubated with lipopolysaccharide (LPS), alone (negative control), OTMC, LCP−1, or OTMC mixed with LCP−1. Three mice were used for biological replicates, and each condition was plated in duplicate. As a control, to ensure that cytokine upregulation was indeed directly stimulated by the compounds tested, the cells were plated in identical conditions and incubated with liposomes for 24 h at 4 °C. Following 24 h incubation with the compounds, the cell supernatant was collected to analyze mouse cytokine expression by ELISA for TNFα (cat. 558534, BD), IL1β (cat. 432601 Biolgened, CA, USA), IL6 (cat. 555240, BD), IL4 (cat. 555232, BD), IL12 (cat. 555256, BD), and IL23 (cat. 433704, Biolegend). ELISA was performed following the manufacturer’s instructions. Absorbance was read on a plate reader (Multiscan GO, Thermo Fisher, Waltham, MA, USA) at 450 nm, within 30 min of adding the stop solution. Background absorbance was subtracted from all data points. Standard curves were generated using either linear regression or four-parameter logistic regression.

### 2.7. Ethics Statement

This study was performed according to the regulations set by the National Health and Medical Research Council (NHMRC) of Australia (Australian Code of Practice for the Care and Use of Animals for Scientific Purposes, 8th edition, 2013). All animal procedures and protocols were approved by The University of Queensland Animal Ethics Committee (AEC), AEC Approval Number SCMB/AIBN/069/17. In addition, C57BL/6 mice 6–8 weeks old were obtained from the Animal Resource Centre (ARC) in Western Australia. All ex vivo experiments had ethics approval by the Research Animal Facility (RAF) of RMIT university under AEC-approved project number 1917.

### 2.8. Statistical Analysis

GraphPad Prism^®^ 7 software (GraphPad Software, Inc., San Diego, CA, USA) was used for all statistical analysis. One-way ANOVA followed by Tukey’s multiple comparison test was applied for statistical analysis, or multiple comparison Kruskal–Wallis tests with *p* < 0.05 were considered statistically significant.

## 3. Results

Liposomes **L1**–**L5** were prepared without extrusion as multilamellar vesicles. Liposomes were composed of neutral lipids (composed of DPPC and CH) and the positively charged (due to high cationic amino acid content) lipopeptide vaccine, LCP−1. They had an overall positive surface charge (50 ± 2 mV). These liposomes carried additional anchored moieties: lipoKALA (**L1**), OTMC (**L2**), lipoPEI (**L3**), or were coated with alginate/DEAE-dextran (**L4**), or alginate/TMC (**L5**). All were positively charged according to DLS analysis. OTMC-bearing liposomes (**L2**) had a higher positive charge than TMC-bearing liposomes (**L5**), which can be explained by the presence of negatively charged alginate in **L5**. **L1**–**L5** formed a variety of sizes, ranging from 60–5000 nm, and were highly polydisperse (PDI), which is typical for multilamellar liposomes (Supplementary Figure S2). Physical mixtures of LCP−1/OTMC, LCP−1/DEAE-dextran, and LCP−1/TMC were also prepared.

**Figure 3:**
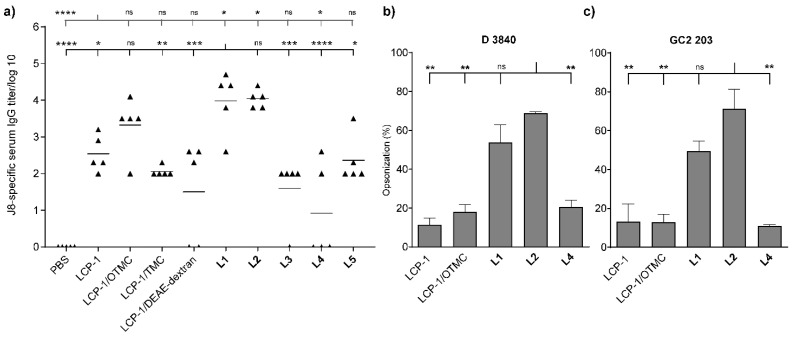
(**a**) J8-specific antibody responses (log10) following intranasal administration of LCP−1-based liposomes and controls in Swiss mice (*n* = 5), as determined by ELISA. Serum was collected on day 52. The average opsonization percentage of group A Streptococcus strains (**b**) D3840 and (**c**) GC2 203. Statistical analysis was performed using one-way ANOVA followed by Tukey’s post-hoc test. All groups were compared with **L1** or LCP−1. Not significant (ns), *p* > 0.05; *; *p* < 0.05; **; *p* < 0.01; ***; *p* < 0.001, and ****; *p* < 0.0001.

The new liposome- and physical mixture-based formulations were examined in outbred Swiss mice for the ability to induce antibody production upon intranasal immunization, and compared to our previous lead formulation, **L1** [21], bearing lipoKALA as an immune enhancer. Again, **L1** elicited a high J8-specific IgG titer, which was significantly higher than those induced by LCP−1 alone (Figure 3a). Interestingly, a comparable J8-specific IgG titer was produced by mice vaccinated with **L2,** which incorporated OTMC. Moreover, when mice were immunized with OTMC and physically mixed with LCP−1, the produced IgG titers were not significantly different from **L1**. In contrast, significantly lower antibody titers were produced by mice vaccinated with the two coated liposomes (**L4** and **L5**). **L3**, which contained lipoPEI, produced a higher IgG titer compared to the negative control (PBS) group, but it was not significantly different from that of mice immunized with LCP−1 alone. Similarly, the two physical mixtures, LCP−1/DEAE-dextran and LCP−1/TMC, did not stimulate higher IgG expression than LCP−1. The serum isolated from mice immunized with the three most effective formulations (**L1**, **L2,** and LCP−1/OTMC). The weakly performing **L4** and LCP−1 (control) were analyzed for their ability to kill GAS bacteria. **L1**, and especially **L2**, had the highest opsonization potentials against both GAS clinical isolates (Figure 3b,c).

**Figure 4:**
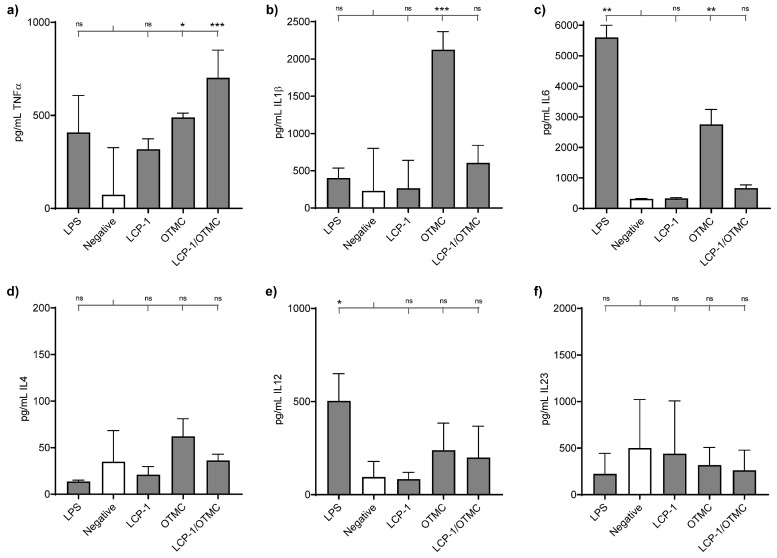
Cytokine production by BMDCs following stimulation with LPS, alone (negative control), OTMC, LCP−1, and OTMC mixed with LCP−1. Cytokine production was expressed as pg/mL ± SD. Production of (**a**) TNFα; (**b**) IL-1β; (**c**) IL6; (**d**) IL4; (**e**) IL12; and (**f**) IL23 was detected following BMDC stimulation by LPS (1 μg/mL). Statistical analysis was performed using multiple comparison Kruskal–Wallis test (ns, *p* > 0.05; * *p* < 0.05; ** *p* < 0.01; and *** *p* < 0.001).

Cytokine profiling was performed using C57BL/6 mouse-derived BMDCs upon treatment with OTMC and controls, as this chitosan derivative had not been previously tested for its adjuvanting capabilities (Figure 4). Secretion of pro-inflammatory cytokine TNFα by BMDCs was significantly higher for LCP−1/OTMC and OTMC than for the negative control (Figure 4a). OTMC also induced significantly higher levels of expression of pro-inflammatory cytokine IL6 and IL1β, while its mixture with LCP−1 did not (Figure 4b,c). While the production of these cytokines was higher in cells treated with OTMC than LCP−1/OTMC, the difference was not statistically significant. Despite its reported self-adjuvanting properties [29], LCP−1, alone, did not induce significant cytokine release with the concentration of tested lipopeptide. Production of IL4 and IL12 was slightly upregulated by LCP−1/OTMC and OTMC; however, this did not differ significantly from the negative control (Figure 4d,e). None of the compounds stimulated the production of IL23 (Figure 4f). The upregulation of cytokine production was not observed under cold conditions (Supplementary Figure S3). Interestingly, TNFα and IL1β production triggered by OTMC was higher than with the gold-standard stimulant, LPS, in the experimental conditions used here.

## 4. Discussion

Liposomal antigen delivery has been recognized as a promising strategy for vaccine development. Liposome-based vaccine formulations have been approved for human use against hepatitis A (Epaxal) and influenza (Inflexal V) [17,30]. Liposomes can carry antigens encapsulated inside their aqueous core or attached to their membrane via lipid-based anchoring. Furthermore, a variety of lipidated moieties can be anchored to liposomes to modify their properties. One such moiety, CPPs, has been used to enhance vaccine efficacy [31]. We recently screened a variety of lipidated CPPs anchored to liposomes for their ability to enhance humoral immune responses against GAS. Among the tested formulations, multilamellar liposomes were the most effective. Of the CPPs tested, lipoKALA (Figure 1) stimulated the production of the highest level of opsonic IgG titers. Thus, the optimized formulation, **L1** carrying lipoKALA, was chosen as a control for the further development of intranasal vaccine delivery systems. For this study, we selected a variety of known and previously examined immune-stimulating moieties (TMC, lipoPEI, DEAE-dextran) [22,26,32], as well as untested OTMC. Chitosan derivatives (e.g., TMC) are known immune stimulators [33], while lipidated sugars often demonstrate adjuvanting abilities, especially once incorporated into liposomes [34,35,36,37,38]. Therefore, we hypothesized that a lipidated analog of TMC (OTMC) may also be an effective immune stimulator.

Multilamellar liposomes were chosen as a vaccine carrier based on our previous studies demonstrating their (i.e., **L1**) ability to induce stronger immune responses compared to unilamellar liposomes (120 nm) [21]. Indeed, we also demonstrated that smaller unilamellar liposomes induced higher antibody titers than larger (70 nm > 140 nm > 400 nm) in mice following intranasal administration; however, multilamellar liposomes (150–1000 nm) were as effective as the smallest unilamellar liposomes [39]. Thus, four new liposomal formulations (**L2**–**L5**) were produced and examined for their ability to stimulate antibody production upon intranasal administration in outbred mice. As expected, liposome **L1** induced high-level antigen-specific IgG titers. Surprisingly, coated liposome **L5** failed to improve the immunogenicity of LCP−1, despite the TMC/alginate system having been reported as effective previously [26,28,40,41]. DEAE-dextran and lipidated PEI were even less effective than TMC.

It is important to note that the polymers mentioned above were examined previously only in unilamellar liposomal formulation, not multilamellar, as reported here. The new chitosan derivative, OTMC, showed a remarkable ability to induce IgG production once formulated into liposomes (**L2**) and was effective even as a simple mixture with LCP−1. However, once antibodies were tested for their GAS opsonization efficacy, only the liposomal formulation, **L2**, triggered the production of clearly opsonic antibodies at the tested concentrations. The high efficacy of **L2** was related to the special properties of OTMC, rather than the high positive charge of the liposomes (e.g., **L2** = +63 mV vs. **L5** = +36 mV), as OTMC was also very immunogenic in the physical mixture (LCP−1/OTMC vs. LCP−1/TMC, Figure 3a). In addition, OTMC/LCP−1 was more immunogenic than TMC/LCP−1, suggesting that more than the liposome-anchoring ability of OTMC was responsible for its activity. Thus, to further analyze the ability of OTMC to act as an adjuvant, we analyzed its ability to stimulate cytokine production. Pro-inflammatory cytokines play an important role in the control of adaptive immune responses. Several different cytokines were tested (TNFα, IL1β, IL6, IL4, IL12, and IL23). Cytokines, such as IL6 and TNFα, have the ability to enhance immune responses against viral infections/vaccines [42,43,44]. Both IL6 and IL12 play important roles in the induction of immune responses against influenza [42]. IL6 is also involved in the terminal differentiation of B-cells [42]. while IL12 and IL23 regulate the differentiation of CD4+ T follicular helper cells, providing support for B-cell generation of high-affinity antibodies and differentiation of B-cells into memory B-cells [45,46]. Three pro-inflammatory cytokines, TNFα, IL1β, and IL6, were secreted in significantly higher amounts upon dendritic cell stimulation with OTMC. OTMC stimulated higher cytokine production alone, compared to LCP−1, suggesting a lack of synergistic effect between these two lipidated compounds. IL6 and TNFα’s ability to stimulate plasma cell longevity was also reported [47], and OTMC clearly upregulated the production of these cytokines. Moreover, IL1β produced at higher levels by OTMC than even the positive control LPS, and has a critical non-dispensable role in stimulating and priming naïve T cell activation [48], a useful new feature for this potential new adjuvant. In addition, cytokines IL12 and IL23 have been associated with autoimmunity and extensive inflammation [49]. Harmful effects associated with the overexpression of IL-4 have also been reported [50]. OTMC did not greatly enhance the level of IL4, IL12, or IL23 produced by BMDCs, suggesting no undesired immunity would be stimulated. Finally, overexpression of IL-1β can be associated with pain, inflammation, and autoimmune reactions [51]. Although OTMC significantly overexpressed this cytokine, we did not observe any adverse effects in mice immunized with OTMC. This could mean that, when administered together with LCP−1, it does not overstimulate IL-1β production.

In summary, both liposomal formulations, **L1** (bearing lipidated CPP KALA) and **L2** (bearing lipidated TMC), stimulated the production of fully functional opsonic antibodies. A newly discovered adjuvant (OTMC) showed the ability to trigger cytokine release by dendritic cells, as well as IgG production, even when administered as a physical mixture with the antigen. However, it was more effective in liposomal formulation.

## 5. Conclusions

We examined a variety of liposomal intranasal vaccine delivery systems. While we confirmed that the lipoKALA liposome delivery strategy is efficient in inducing antibody production, we also discovered the remarkable adjuvanting capacity of OTMC. OTMC was not only effective in enhancing antibody production upon anchoring to liposomes, but also on its own when mixed with the LCP−1 vaccine. OTMC triggered cytokine release by dendritic cells when administered with or without LCP−1, further confirming its adjuvanting activity. TMC/DEAE dextran-coated and lipoPEI-incorporated liposomes were much less immunogenic than expected. In summary, liposomes bearing lipoKALA and OTMC are promising self-adjuvanting platforms for intranasal peptide-based vaccine delivery.

## Data Availability

The data presented in this study are available in the article and Supplementary Materials.

## Supplementary Materials

The following are available online at https://www.mdpi.com/article/10.3390/vaccines11020305/s1, Figure S1: Coating of polyelectrolyte-coated liposomes. Effect of concentration of sodium alginate, TMC, and DEAE-dextran on zeta potentials of LCP−1 loaded liposome formulation. Zeta potential changes during the coating of liposome with (a) sodium alginate; (b) DEAE-dextran; and (c) with TMC. The red box marks the values selected for the final liposome coating. Figure S2: DLS spectra of particles of (a) **L1**, (b) **L2**, (c) L3, (d) **L4**, and (e) **L5**. Size distributions by intensity, PDI, and zeta potential. Figure S3: Production of (a) TNFα; (b) IL-1β; (c) IL6; (d) IL4; (e) IL12; and IL23 cytokines following stimulation of BMDC in cold-controlled conditions (4 °C). Stimulation was performed with LPS, none (negative control), OTMC, LCP−1, and OTMC mixed with LCP−1. No significant difference between the groups was detected.
