# Supplementary material for: The Development of Surface-Modified Liposomes as an Intranasal Delivery System for Group A Streptococcus Vaccines
Source: Vaccines (Basel). 2023 Jan 30;11(2):305. doi: 10.3390/vaccines11020305 (PMC9961534; doi:10.3390/vaccines11020305)
Supplement: Supplementary file 1 [file vaccines-11-00305-s001.zip › vaccines-2154751-supplementary.pdf]

The Development of Surface-Modified Liposomes as an Intranasal Delivery System for Group A Streptococcus Vaccines

Jieru Yang [1], Jennifer C. Boer [2], Mattaka Khongkow [3], Sarunya Phunpee [3], Zeinab G. Khalil [4], Sahra Bashiri [1], Cyril Deceneux [2], Georgia Goodchild [2], Waleed M. Hussein [1], Robert J. Capon [4], Uracha Ruktanonchai [3], Magdalena Plebanski [2], Istvan Toth [1,4,5] and Mariusz Skwarczynski [1,*]

[1] School of Chemistry and Molecular Biosciences, The University of Queensland, St. Lucia, QLD 4072, Australia
[2] School of Health and Biomedical Sciences, RMIT University, Bundoora, Melbourne, VIC 3083, Australia
[3] National Nanotechnology Center (NANOTEC), National Science and Technology Development Agency (NSTDA), 111 Thailand Science Park, Phahonyothin Road, Klong 1, Pathumthani 12120, Thailand
[4] Institute for Molecular Bioscience, The University of Queensland, St. Lucia, QLD 4072, Australia
[5] School of Pharmacy, The University of Queensland, Woolloongabba, QLD 4102, Australia
* Correspondence: m.skwarczynski@uq.edu.au; Tel.: +61-73-346-9894

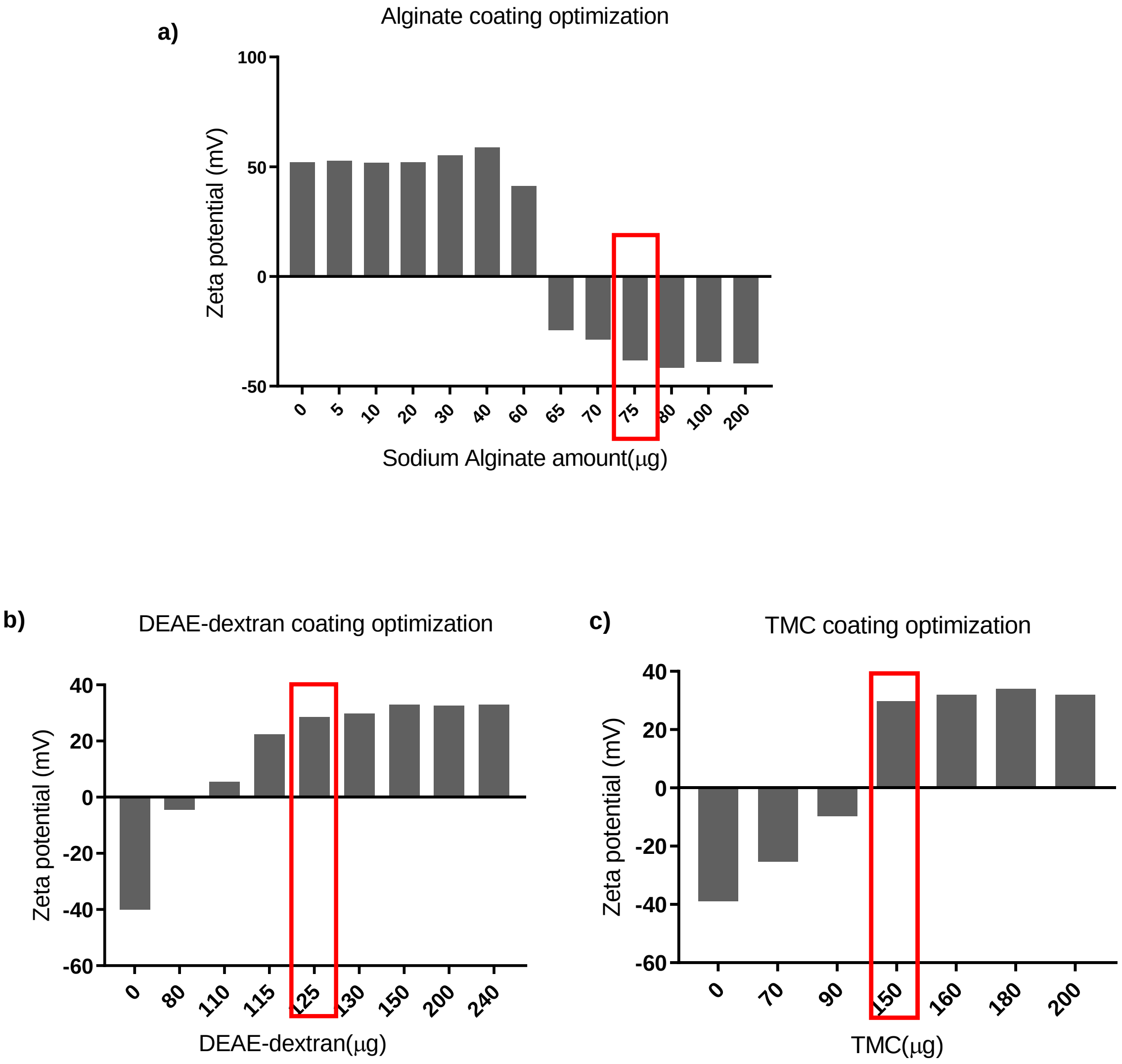

Figure S1. Coating of polyelectrolyte-coated liposomes. Effect of concentration of sodium alginate, TMC and DEAE-dextran on zeta potentials of LCP-1 loaded liposome formulation. Zeta potential changes during coating of liposome with a) sodium alginate; b) DEAE-dextran; c) with TMC. Red box mark values selected for final liposomes coating.

a)

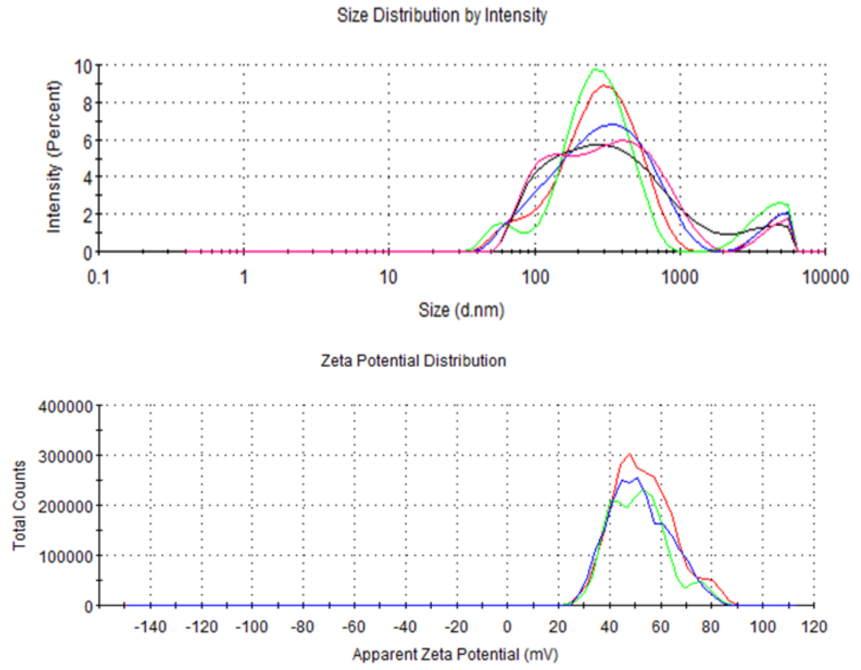

Liposomes	Coating/anchored moieties	Particle size (nm)	Polydispersity index (PDI)	Zeta potential (mV)
**L1**	lipoKALA	Peak 1: 96 ± 45<br>Peak 2: 379 ± 83<br>Peak 3: 4273 ± 286	0.42 ± 0.02	57 ± 2

b)

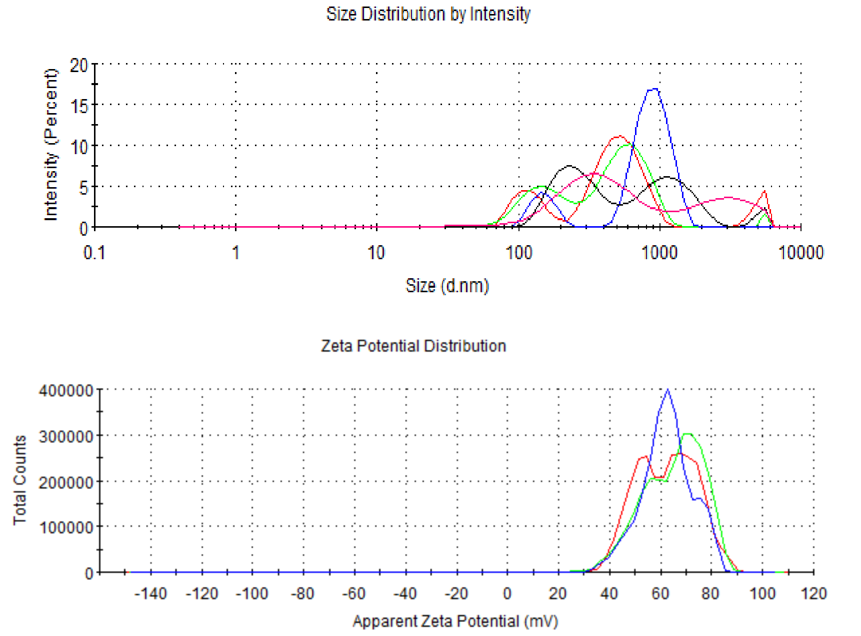

Liposomes	Coating/anchored moieties	Particle size (nm)	Polydispersity index (PDI)	Zeta potential (mV)
**L2**	OTMC	Peak 1: 250 ± 227<br>Peak 2: 641 ± 421<br>Peak 3: 4645 ± 1202	0.51 ± 0.04	63 ± 2

c)

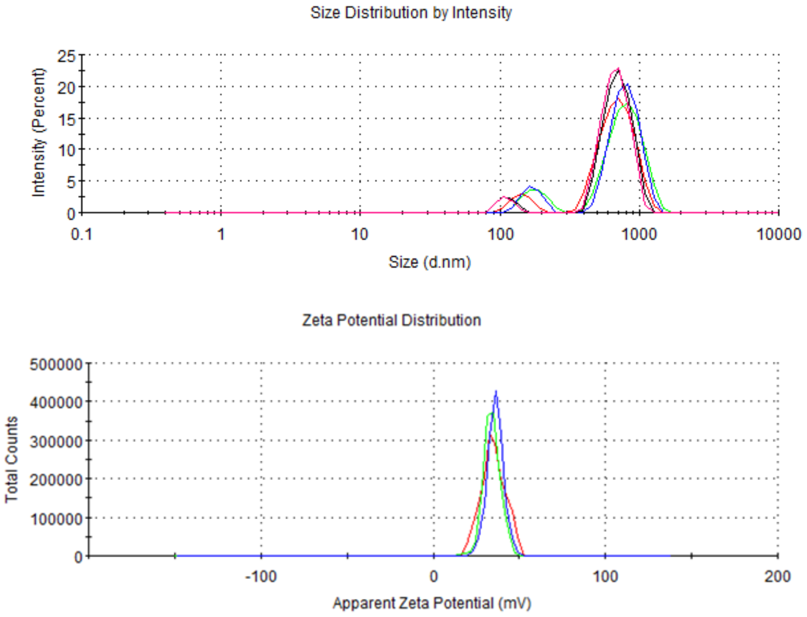

Liposomes	Coating/anchored moieties	Particle size (nm)	Polydispersity index (PDI)	Zeta potential (mV)
**L3**	lipoPEI	Peak 1: 184 ± 116<br>Peak 2: 694 ± 156	0.61 ± 0.03	63 ± 2

d)

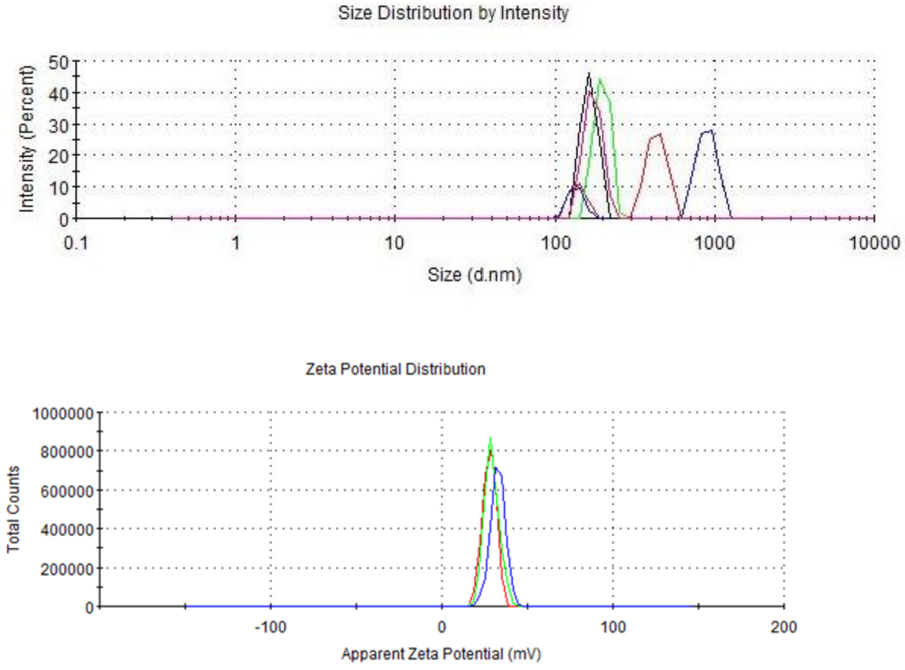

Liposomes	Coating/anchored moieties	Particle size range (nm)	Polydispersity index (PDI)	Zeta potential (mV)
**L4**	alginate/DEAE-dextran	Peak 1: 164 ± 26<br>Peak 2: 667 ± 329	0.46 ± 0.33	30 ± 2

e)

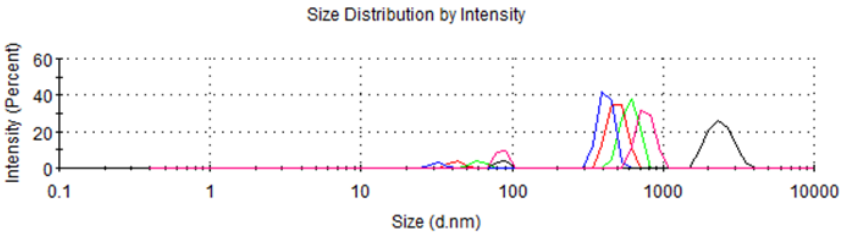

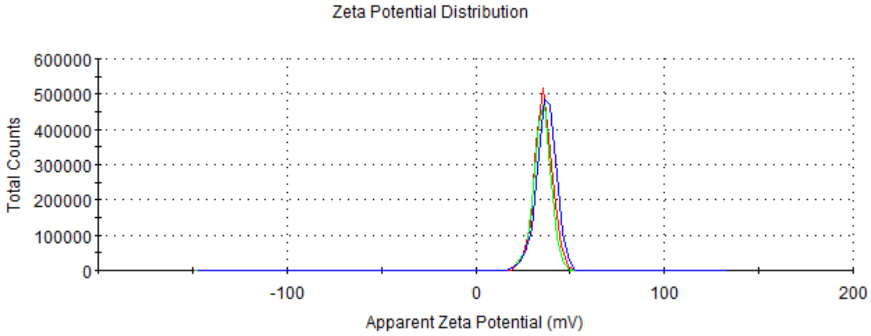

Liposomes	Coating/anchored moieties	Particle size range (nm)	Polydispersity index (PDI)	Zeta potential (mV)
**L5**	alginate/TMC	Peak 1: 61 ± 25<br>Peak 2: 935 ± 824	0.64 ± 0.13	36 ± 1

Figure S2. DLS spectra of particles of a) **L1**, b) **L2**, c) **L3**, d) **L4**, e) **L5**. Size distributions by intensity, PDI and zeta potential.

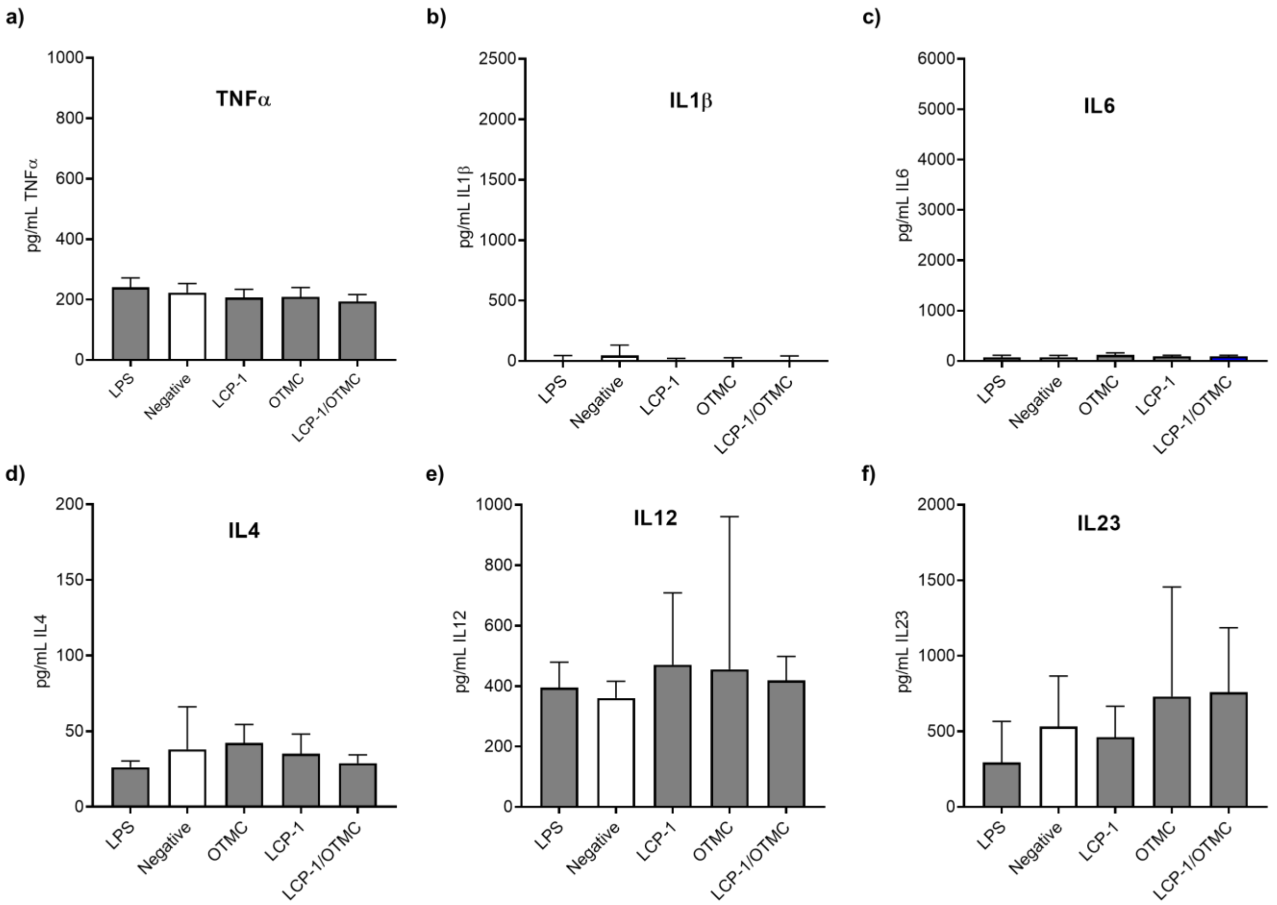

Figure S3. Production of a) TNFα; b) IL-1β; c) IL6; d) IL4; e) IL12; and IL23 cytokines following stimulation of BMDC in cold controlled conditions (4°C). Stimulation was performed with LPS, none (negative control), OTMC, LCP-1, and OTMC mixed with LCP-1. No any significant difference between the groups were detected.
